# SPARC expression in CML is associated to imatinib treatment and to inhibition of leukemia cell proliferation

**DOI:** 10.1186/1471-2407-13-60

**Published:** 2013-02-05

**Authors:** Cesarina Giallongo, Piera La Cava, Daniele Tibullo, Ignazio Barbagallo, Nunziatina Parrinello, Alessandra Cupri, Fabio Stagno, Carla Consoli, Annalisa Chiarenza, Giuseppe A Palumbo, Francesco Di Raimondo

**Affiliations:** 1 Department of Clinical and Molecular Biomedicine, University of Catania, Ospedale Ferrarotto, V. Citelli 6, 95124, Catania, Italy; 2 Department of Biological Chemistry, Medical Chemistry and Molecular Biology, University of Catania, Catania, Italy

**Keywords:** CML, Imatinib, SPARC, Granulocytes, Monocytes

## Abstract

**Background:**

SPARC is a matricellular glycoprotein with growth-inhibitory and antiangiogenic activity in some cell types. The study of this protein in hematopoietic malignancies led to conflicting reports about its role as a tumor suppressor or promoter, depending on its different functions in the tumor microenvironment. In this study we investigated the variations in SPARC production by peripheral blood cells from chronic myeloid leukemia (CML) patients at diagnosis and after treatment and we identified the subpopulation of cells that are the prevalent source of SPARC.

**Methods:**

We evaluated SPARC expression using real-time PCR and western blotting. SPARC serum levels were detected by ELISA assay. Finally we analyzed the interaction between exogenous SPARC and imatinib (IM), in vitro, using ATP-lite and cell cycle analysis.

**Results:**

Our study shows that the CML cells of patients at diagnosis have a low mRNA and protein expression of SPARC. Low serum levels of this protein are also recorded in CML patients at diagnosis. However, after IM treatment we observed an increase of SPARC mRNA, protein, and serum level in the peripheral blood of these patients that had already started at 3 months and was maintained for at least the 18 months of observation. This SPARC increase was predominantly due to monocyte production. In addition, exogenous SPARC protein reduced the growth of K562 cell line and synergized in vitro with IM by inhibiting cell cycle progression from G1 to S phase.

**Conclusion:**

Our results suggest that low endogenous SPARC expression is a constant feature of BCR/ABL positive cells and that IM treatment induces SPARC overproduction by normal cells. This exogenous SPARC may inhibit CML cell proliferation and may synergize with IM activity against CML.

## Background

CML is a myeloproliferative disease caused by the t(9;22) translocation [1] that generates BCR-ABL, a constitutively active tyrosine kinase (TK). IM, a TK inhibitor (TKI), is the elective drug for CML treatment. CML patients in the chronic phase treated with IM achieve deep and durable responses [2]. However, a small percentage of these patients and most advanced-phase patients develop resistance to TKI therapy [3,4].

Secreted protein, acidic and rich in cysteine (SPARC) is a multifunctional matricellular glycoprotein, also known as osteonectin or BM-40. This protein has counter-adhesive properties, has effects on cell shape, immune surveillance and angiogenesis [5]; inhibits cell proliferation and delays cell cycle in the G1 phase [6]. SPARC seems to inhibit cell proliferation after digestion by MMP-3 [7] and links with cell-surface receptors to activate G-protein coupled signaling [8]. SPARC binds VEGF, preventing VEGF-induced tyrosine phosphorylation of VEGFR1 and antagonizing its pro-angiogenic effects [9]. The protein also binds PDGF-B, blocking the binding to its receptors and the proliferation signaling [10].

The role of SPARC in tumor pathogenesis and progression seems to depend on its different functions in the tumor microenvironment. In some types of cancer, SPARC correlates with poor prognosis (melanoma, glioma, prostate and breast cancer), while in others the protein functions as a tumor suppressor (ovarian and colorectal cancers) [11]. In hematological diseases, SPARC has been evaluated on MDS 5q-syndrome and acute myeloid leukemia (AML) with MLL rearrangements. In 5q-MDS, the deletion of SPARC is associated with the pathogenesis of disease and patients responsive to lenalidomide show an increase of SPARC expression [12]. SPARC is transcriptionally silenced in AML with rearrangement of the MLL (Mixed lineage leukemia) gene and may function as a tumor suppressor in this subset of patients. SPARC silencing is associated with promoter methylation in MLL cell lines but not in patients’ cells and the addition of exogenous purified protein inhibits cell line proliferation [13]. In contrast, the SPARC gene was up-regulated in multiple myeloma and plasmacytoma [14]. A recently published study reported that in CML, SPARC accumulates in TKI-resistant CML cell lines. It activates the Fyn/ERK kinase signaling that inhibits apoptosis and promotes IM resistance [15]. In contrast to this work, Li and co-workers [16] showed that transfection of K562 with the SATB1 plasmid induces SPARC over-expression, resulting in a reduction of cell proliferation.

In this study we investigated the variations in SPARC production by peripheral blood cells from CML patients at diagnosis and after treatment and we identified the subpopulation of cells that are the prevalent source of SPARC.

## Results

### SPARC is downregulated in CML cells

**Figure 1:**
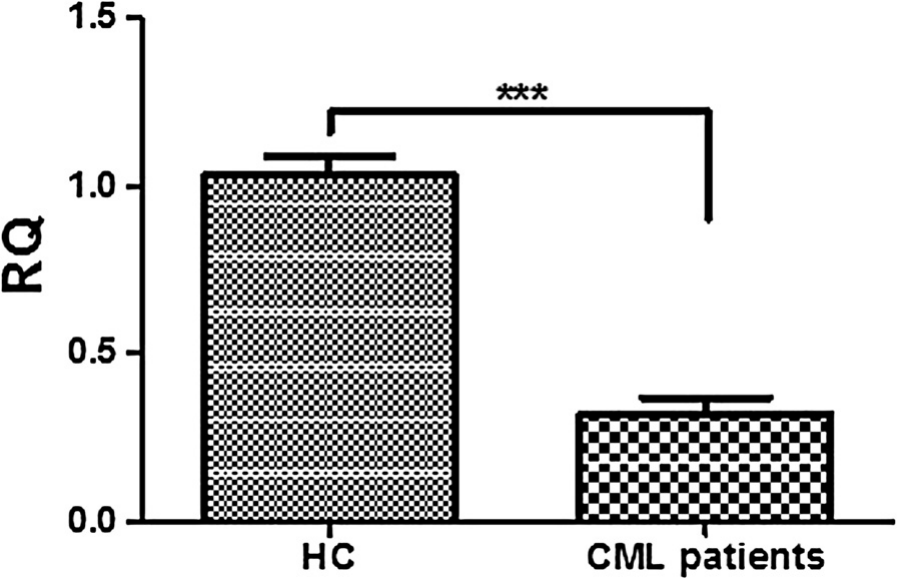
**SPARC is underexpressed in CML patients at diagnosis. Expression of SPARC was analyzed in PBMCs of 20 HC vs 40 CML patients by qRT-PCR. **DATA are expressed as means ± S.D.

**Figure 2:**
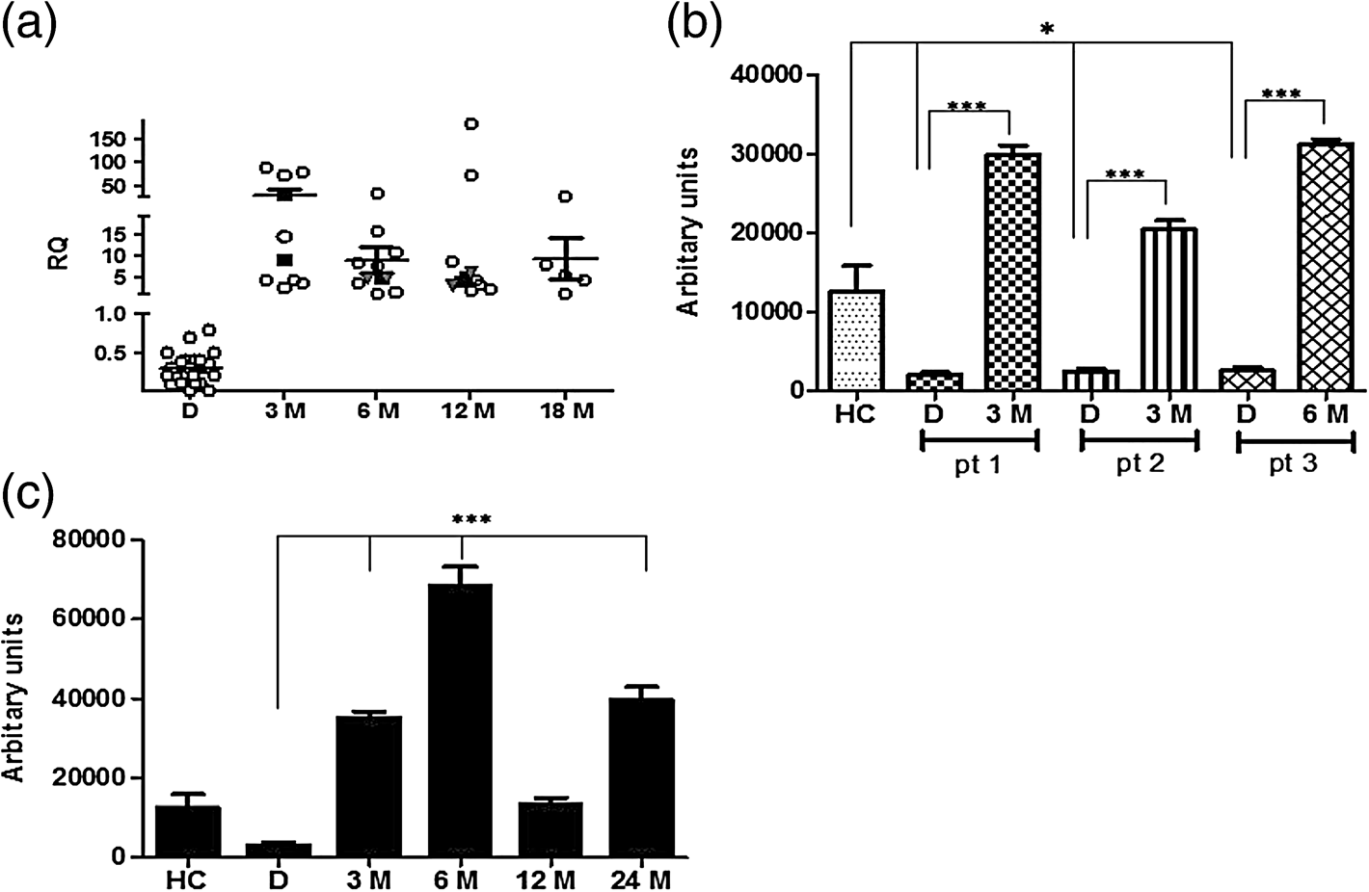
**SPARC expression increased during TKIs therapy. (a) **Expression of SPARC was performed by qRT-PCR in PBMCs of 12 CML patients at diagnosis (D) and during TKIs treatment; D vs 12 M: p<0.002. White circles: IM-treated patients; black squares: NI-treated patients; gray triangles: alternating NI/IM treated patients. Results are expressed with respect to HC. **(b) **Analysis of SPARC expression in PBMCs of 3 CML patients (pt) at D and during IM therapy by western blotting. The optical density of the bands was measured using Scion Image software. Results represent the mean of three indipendent experiments; error bars denote S.D. **(c) **Protein levels decreased after interruption of IM therapy (12 M) and increased after IM was restarted (24 M). Results represent the mean of three indipendent experiments; error bars denote S.D.

SPARC mRNA and protein in CML cells from patients at diagnosis was downregulated with respect to healthy controls (HC) (p<0.001 for mRNA and p<0.05 for protein) (Figures 1 and 2b). Twenty-two patients were evaluated during TKI treatment: IM, NI (Nilotinib) or alternating IM and NI every three months. In all TKI treated patients a significant increase of SPARC mRNA expression was recorded at 3 months of treatment and this increase was maintained at 18 months of therapy (p<0.01) (Figure 2a). A similar increase of the SPARC protein level was observed in the peripheral blood cells of three analyzed patients (p<0.001) during IM treatment (Figure 2b). Interestingly, in one patient, discontinuation of IM at the 12^th^ month, resulted in a significant decrease of the SPARC protein level that increased again after IM was restarted (Figure 2c).

**Figure 3:**
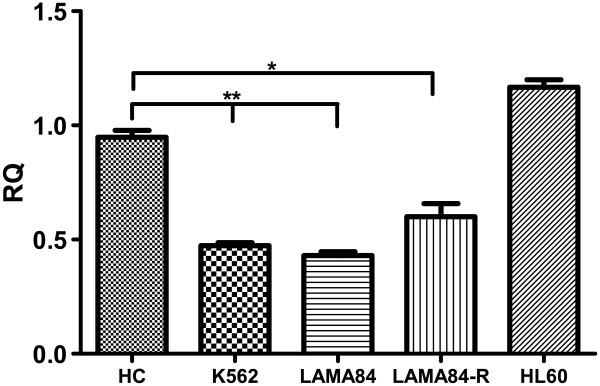
**Quantitative real time RT-PCR analysis of SPARC expression in myeloid leukemia cell lines. **Bars represent the mean and standard deviation of three independent experiment. SPARC resulted significantly down-regulated in BCR/ABL positive cell lines only. K562 and LAMA84 vs HC: p<0.01; LAMA84-R vs HC: p<0.05.

We also measured SPARC level in BCR/ABL positive cell lines (K652, LAMA84) and in HL60, a BCR/ABL negative acute myeloid leukemia cell line (Figure 3). The protein appears to be downregulated only in chronic myeloid leukemia cell lines while no difference of SPARC expression was observed in HL60 in respect to HC.

### IM increases SPARC levels in the sera of patients

**Figure 4:**
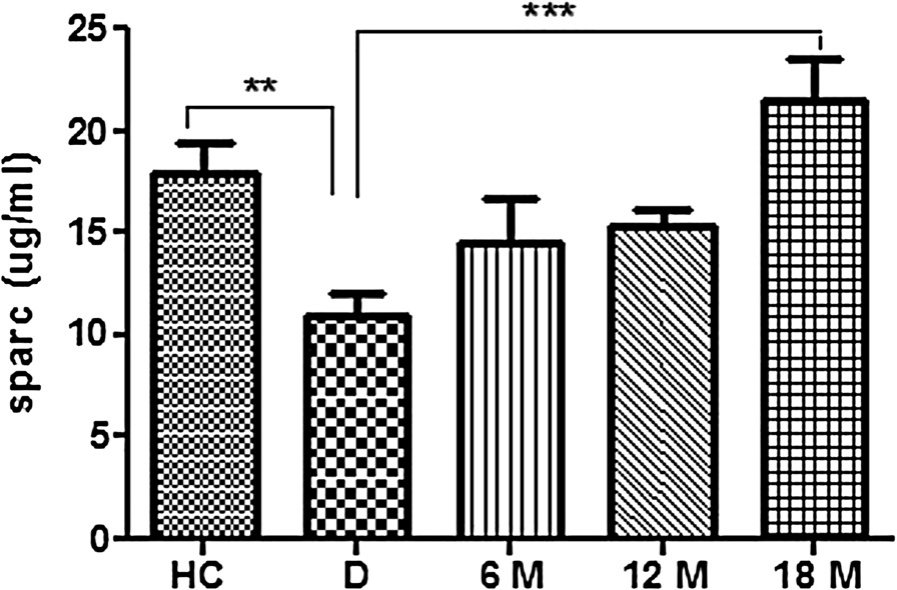
**Levels of secreted SPARC increased during IM therapy. **Secreted protein was evaluated by ELISA in CML 10 patients at D, 6 of which followed during TKI therapy. Data are expressed as means ± S.D.

We also evaluated the secreted form of SPARC in 10 CML patients at diagnosis and in 6 of them at 6, 12, and 18 months during IM treatment by ELISA. We found that SPARC serum levels were decreased at diagnosis compared to HC (p<0.01) but then increased progressively, achieving normal levels at 18 months (p<0.001) (Figure 4).

### In lymphoid cells, monocytes and granulocytes from CML patients, SPARC was downregulated at diagnosis but increased during IM therapy

**Figure 5:**
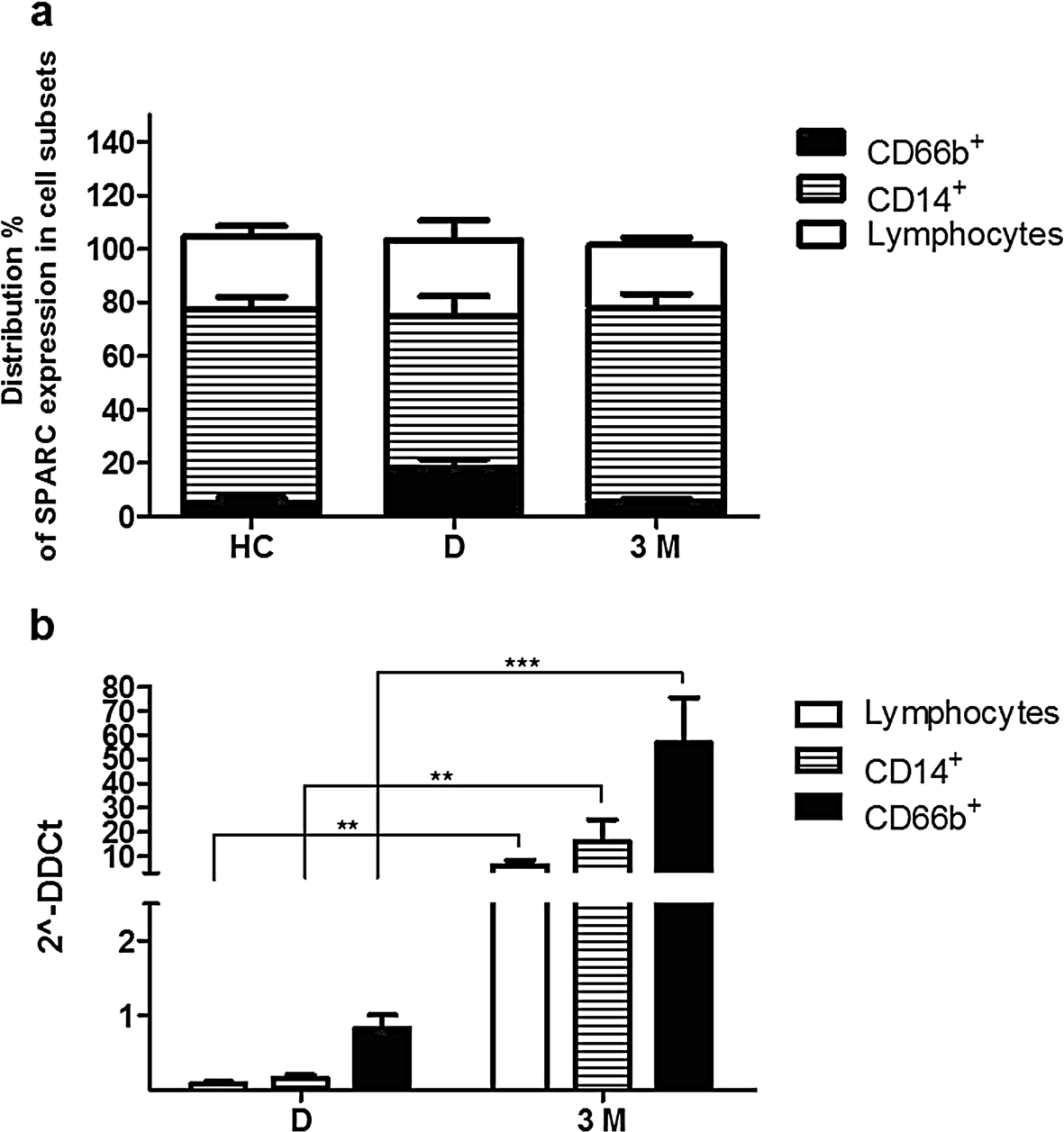
**SPARC mRNA expression by qRT-PCR in cell subsets after magnetic separation tecnology. a. **Distribution of SPARC expression in PBMCs of 4 HD and 4 CML patients at D and after 3 M of IM therapy calculated as percentage of expressing SPARC granulocytes, monocytes and lymphocytes in respect to the total number of separated expressing SPARC cells. Data are expressed as means ± S.D. **b. **SPARC mRNA levels in cell subsets of CML patients at D and after 3 M of therapy compared with the same cell populations of HC (calculated value of 2^-ΔΔCt in HD’ subsets was 1). Data are expressed as means ± S.D.

In order to test which cell population predominantly expresses SPARC mRNA, we separated PBMCs of 4 HC and 4 CML patients, the latter at diagnosis and after 3 months of IM therapy. After magnetic separation of lymphoid cells (CD3^+^ and CD19^+^), granulocytes (CD66b^+^) and monocytes (CD14^+^), we tested the expression of SPARC mRNA in each of these subsets. As shown in Figure 5a, SPARC was predominantly expressed in monocytes with respect to the total number of separated cells both in HC and in CML patients. In the analyzed cell subsets there was a similar distribution of SPARC expression for HC and treated patients groups while in CML patients at diagnosis an increase of granulocytes expressing SPARC was observed at the expense of monocytes. SPARC mRNA expression was calculated in each sub-population of patients at diagnosis and after 3 months of IM treatment and compared with the correspondent subsets of HC (Figure 5b). After IM treatment, all 4 studied patients were in complete hematological remission, with a 87±0.3% reduction of BCR/ABL transcript.

SPARC expression in every cell population of CML patients at diagnosis was lower than HC (lymphoid cells: 12 fold; monocytes: 9 fold; granulocytes: 2 fold), while after 3 months of IM treatment an increase of SPARC mRNA was found in all cell sub-populations (lymphoid cells: 6 fold; monocytes: 16 fold; granulocytes: 57 fold compared to HC cell subsets), (diagnosis vs treatment: p<0.01 in lymphoid cells and monocytes; p<0.001 in granulocytes).

These results confirm that granulocytes from CML patients at diagnosis express low levels of SPARC mRNA and they also suggest that the percentage of normal cells that express SPARC mRNA was lower than HC, thus justifying our finding of low SPARC levels in CML PBMC at diagnosis. On the contrary, distribution of SPARC expression in normal cell populations exceeded that of HC during IM treatment, indicating that the increase of SPARC mRNA levels that we observed in PBMC during IM treatment is mainly due to normal cells and in particular to normal monocytes.

In conclusion, the observed increase in SPARC expression after IM therapy is due to increased production by non-neoplastic cells such as lymphocytes, monocytes and granulocytes. The last population showed the highest increase but it should be underlined that they are actually two different populations since at diagnosis most granulocytes belong to the neoplastic clone while at 3 months most granulocytes are normal.

### SPARC inhibits the growth of K562 cell line

**Figure 6:**
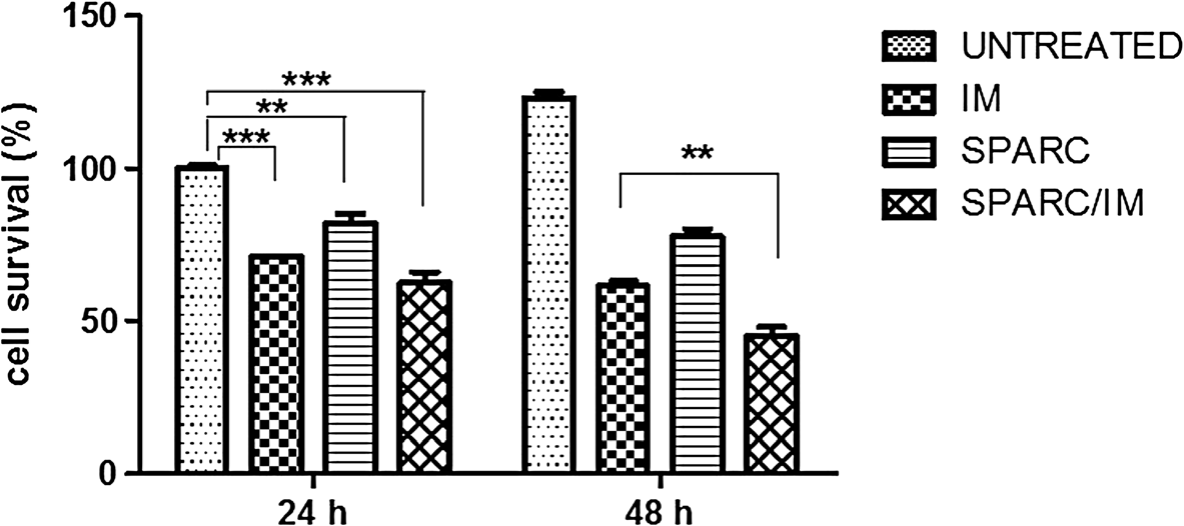
**The graph is representative for K562 cell line. **Cell survival after exposure to IM, SPARC or their combination is showed. Cells were pre-treated with SPARC followed by IM for 24 h. Percentage of viable cells was measured by ATP.lite at 24 h and 48 h after addition of IM. Results represent the means of five independent experiments; error bars denote S.D.

SPARC has been shown to induce growth arrest and apoptosis in a number of different cell lines [13,17]. We hypothesized that K562 and LAMA84 cells might also be sensitive to these SPARC effects. To prove this hypothesis, we first investigated whether SPARC increase during therapy might contribute to TKIs cytotoxicity. K562 cells were treated with serum before and after IM therapy (6 months) of 6 different patients. In all conditions with serum during therapy, K562 showed a mortality between 25-31±9% in respect to the same sample at diagnosis. Anyhow SPARC antibody didn’t induce any change in our results. Therefore, in these experiments, the activity of IM is not mediated by circulating SPARC. Second, we exposed cells to human recombinant SPARC 2 days before exposure to IM, so that the protein was efficiently internalized in the cells. After 72 h we evaluated cell proliferation and cell cycle progression. SPARC alone and IM alone induced a reduced survival rate of 18±3*.*2% and 29±1*.*6% respectively vs untreated cells; their association led to a reduced cell viability of 37*.*5±3*.*7% (Figure 6). After 96 h of incubation the effect of the combination SPARC/IM was more evident since it induced a reduction of viability that was 16*.*5±3% higher than that induced by IM alone (p<0.01).

**Figure 7:**
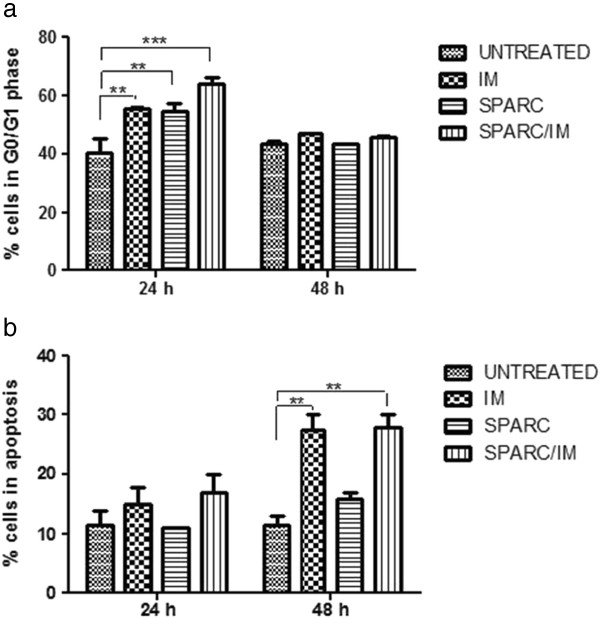
**Effect of IM, SPARC and their combination on the G0/G1 phase and apoptosis in K562 cells. **Cell cycle distribution were assessed at 24 (**a**) and 48 h (**b**) after cell exposure to IM. Analysis was performed by the ModFit program. Results represent the means of five indipendent experiments; error bars denote S.D.

We next evaluated cell cycle induced modifications and, as expected, IM inhibition of BCR/ABL tyrosine kinase activity resulted in a G1 cell cycle arrest [15,18,19]. Flow cytometry revealed an accumulation of K562 cells in G0/G1 24 h after IM exposure (15±1*.*7% more than untreated cells; p<0.01) (Figure 7a). Recombinant SPARC alone also induced the same block of the cell cycle (14*.*5±4*.*1% more than untreated cells; p<0.01) and the combination SPARC/IM showed an additive effect (26*.*5±3*.*3% more than untreated cells; p<0.001). This cell cycle arrest was not recorded after 48 h of IM exposure while at this time point an increase of apoptosis was observed both in IM and SPARC/IM conditions (respectively of 16±4% and 16*.*5±3*.*7% vs untreated cells; p<0.01), but not with SPARC alone (Figure 7b). Similar data were obtained in LAMA84-S cell line. On the contrary, LAMA 84-R, resistant to IM by mechanism of gene amplification, did not show any cell cycle arrest after exposure to exogenous SPARC. Therefore, the recombinant protein did not sensitize LAMA84-R to IM both at 24 and 48 hs of SPARC and TKI combination exposure (data not shown).

In addition we analyzed effect of exogenous SPARC on HL60 cells line to examine if recombinant protein leads to a cell cycle arrest as in BCR/ABL positive cells sensible to IM. For this evaluation, we used the same concentration (15 μg/ml) used in K562 and in LAMA84 cell lines. As assumed by real time PCR data, the protein did not induce any variation in cell cycle of HL60 in comparison to untreated cells (data not shown).

These results suggest that exogenous SPARC is not able to induce apoptosis in CML cells but it inhibits progression from the G1 to S phase in K562 and LAMA84 sensible to IM and its combination with IM potentiates the TKI effect on the cell cycle.

## Discussion

In this paper we show that CML cells have a low level of SPARC and there is also a reduction of the secreted form of SPARC in the serum of CML patients. Once treatment with IM was initiated, we observed an increase of SPARC mRNA and protein in the PBMCs, reaching levels that are much higher than normal controls. This increase was evident already at 3 months and was maintained at least for the 18 months of observation. Accordingly, the concentration of SPARC in the serum showed a progressive increase during IM treatment and reached normal values at 18 months. In fact, IM is a very effective treatment for CML. At 3 months, most patients are in complete hematological response and at 12 months most of them are in complete cytogenetic or even molecular response. In these conditions, CML cells are virtually absent in the PBMCs of CML patients and, however, the contribution of these cells to the PBMC composition is negligible. Therefore, the measured SPARC mRNA after 3 months of treatment and throughout the subsequent evaluations was produced by normal cells, i.e. lymphoid cells, monocytes and non-clonal granulocytes. This production is probably favored by IM since we observed that, in one patient, discontinuation of IM therapy resulted in a reduction of the SPARC level while it rose again after restarting treatment. The only two patients treated with an alternation of IM and NI, showed the same pattern as patients treated with IM alone, thus favoring the hypothesis that the increase of SPARC is a common phenomenon after any TKI treatment.

We show that SPARC is mainly expressed by monocytes with respect to the total number of analyzed cells but, during IM treatment, other cell populations such as normal lymphocytes and granulocytes increase their SPARC production.

We also demonstrated that SPARC may synergize in vitro with IM by blocking the cell cycle of CML cells in the G0/G1 phase. We therefore hypothesize that the increased production of SPARC by normal cells may contribute to the efficacy of IM in reducing CML clone. In this perspective, studies are ongoing in our lab in order to evaluate if the levels of SPARC that can be achieved after treatment may be correlated with the magnitude of the response to IM.

The study of SPARC in hematopoietic malignancies led to conflicting reports about its role as a tumor suppressor or promoter. A study by Li et al. supports an anti-proliferative effect of SPARC on leukemic cells. The authors reported that transfection of K562 cells with SATB1 plasmid induced SPARC over-expression, resulting in a reduction of cell proliferation [16]. In contrast, a recent study [15] reports intracellular SPARC as a potential cause of resistance to IM in CML cells. However, the same authors pointed out that the cellular localization of SPARC could be determinant in predicting its activity. The discrepancy that exists in the literature on the activity of SPARC on different diseases is probably linked to the fact that there are different sources and different isoforms and even in the same disease SPARC may have different activities. In addition, the activity of SPARC may depend on whether it is secreted and associated with the extracellular matrix or retained inside the cell. In this perspective, if the intracellular form of SPARC may protect CML cells from IM induced cell death [15], its secreted form could even be toxic for the CML cells as it has been demonstrated not only by our experiments but also for some subtypes of acute myeloid leukaemia [13].

Initial studies showed that SPARC is important in bone mineralization [20]. In this perspective, we [21] and others [22-25] have demonstrated that in CML patients treated with IM there is a transient increase in bone-formation markers that could be linked to inhibition of PDGFR signaling. We have also demonstrated that also NI induced osteoblastogenesis in vitro, probably triggering the same IM targets [26]. Therefore the increase of SPARC induced by TKIs could be related to the well documented modification of the bone metabolism observed in IM treated patients.

In CML cells, the BCR/ABL oncoprotein has proliferative effects activating the Ras/Raf/MEK/ERK, JAK/STAT and PI3K/Akt pathways [27-30]. The autophosphorylation site Y177 on BCR/ABL binds the scaffolding adaptor protein Gab2 [28] that activate both PI3K/Akt and Raf/MEK/ERK pathways [27]. Inhibition of BCR/ABL by IM results in a G1 cell cycle arrest mediated by the PI3K pathway [31]. One method that is showing success against IM-resistant CML cells is the treatment with inhibitors that target Ras or PI3K [32]. Because SPARC affects multiple downstream signaling pathways, such as PI3K/AKT [11], in our study we focused on its potential role as a tumor suppressor protein in CML cells. Many studies have focused on the role of the protein in tumorigenesis, but few on its potential role in modulating therapy sensitivity [33]. Here, we demonstrate that exogenous exposure to SPARC in K562 cells induced a G0/G1 cell cycle arrest reducing the growth rate of these cells and appeared to confer an increased sensitivity of K562 cells to IM. In addition, residual leukemia stem and progenitor cells persist in IM-responsive patients and may be a potential source of relapse. While integrins such as very late antigen-4 and the adhesion molecule CD44 have been demonstrated to be crucial for the persistence of leukemic progenitors in the bone marrow [34], SPARC is an anti-adhesive molecule and modulates the cell matrix [35]; its down-regulation may increase the adhesion of the hematopoietic stem cells to the supporting stromal cells and provide a clonal advantage [36]. Therefore the SPARC/TKI association could serve to enhance the TKI effect on leukemic stem cells that are not eliminated by BCR/ABL inhibitors [37-39].

On the basis of 1) our demonstration that in vitro SPARC has an anti-proliferative effect on BCR/ABL positive cells and influences the sensitivity of leukemic cells to therapy and 2) the putative modulation of the tumor microenviroment by this protein, it is conceivable that the effects of exogenous SPARC could be exploited therapeutically by using recombinant SPARC or one of its derivate peptides. This possible approach is supported by experiments on xenograft tumor models where intraperitoneal or subcutaneous injections of SPARC inhibited tumor growth [33,40].

## Conclusion

Our data demonstrate that endogenous SPARC is reduced in CML cells but IM treatment induces an increased production of exogenous SPARC by normal cells, mainly monocytes; since this protein has an anti-proliferative effect, it may even contribute to the activity of IM on leukemic cells.

## Methods

### RNA extraction and qRT-PCR

Peripheral blood mononuclear cells (PBMCs) of patients at diagnosis and during TKI treatment were obtained using a Ficoll-Hypaque gradient. We analyzed 40 CML patients at diagnosis, and for 22 of them we also collected samples during first-line treatment with IM (18 patients), nilotinib (2 patients) or alternating treatment with nilotinib and IM every three months (2 patients). All enrolled patients signed an informed consent form and all were followed with a monthly CBC count, molecular evaluation of the BCR/ABL transcript every 3 months, and cytogenetic evaluation every 6 months, according to ELN guidelines.

RNA was extracted by Trizol reagent (Invitrogen, Carlsbad, CA, USA). First strand cDNA was then synthesized with Applied Biosystem (Foster City, CA, USA) reverse transcription reagent [41].

SPARC mRNA expression was assessed by TaqMan Gene Expression, Applied Biosystem and quantified using a fluorescence-based real-time detection method by LightCycler (Roche Diagnostic Corp., Indianapolis, IN, USA). For each patient, the relative expression level of SPARC mRNA was normalized using ABL as an invariant control.

### Western blot analysis

Western Blot Analysis was performed according to the manufacturer’s recommendations [42]. The antibody directed against the human SPARC was obtained from Haematological Thecnologies. An anti-mouse antibody against actin (Sigma, St. Louis, MO, USA) was used to assess equal loading. The blots were scanned, and the optical density of the bands was measured using Scion Image software (New York, NY).

### ELISA assay

By using a specific enzyme-linked immunosorbent assay (ELISA) test (R&D Systems, Abingdon, United Kingdom), we evaluated SPARC levels in the serum of patients both at diagnosis and at different time points during treatment with IM. Ten healthy volunteers were used as control.

### Lymphoid cell, granulocyte and monocyte separation

Purification of lymphoid cells (CD3^+^ and CD19^+^), granulocytes (CD66b^+^) and monocytes (CD14^+^) from PBMCs was performed by a positive selection of these cells using a magnetic separation kit (EasySep, STEMCELL Technologies, Vancouver, BC, Canada). Cell purity was determined by flow cytometry and was >95% for each cell population.

### Cells and culture conditions

K562, LAMA84 and LAMA84-R (resistant to IM) cell line were cultured at 37°C in 5% CO_2_ in RPMI-1640 (CELBIO) with 10% FBS (Fetal Bovine Serum) and 1% penicillin-streptomycin. For HL60 cells we used DMEM (CELBIO) with 20% FBS and 1% penicillin-streptomycin.

In vitro SPARC treatment was performed by adding 15 μg/ml exogenous SPARC (R&D system) for 48 h followed by 24 h of IM (Novartis Farma Spa, Origgio, Italy) 1 μM. Vitality and cell cycle distribution were assessed at 24 and 48 h after cell exposure to IM.

### ATP-lite1step assay for cell survival

The viability of cells was evaluated by the ATP-lite1step assay (PerkinElmer, Monza, Italy). Luminescence was measured using a Victor3 reader (Perkin Elmer).

Cell survival was calculated as the percentage of viable cells in the treated suspension culture compared to the untreated one.

### Cell cycle analysis

Cells were washed and resuspended in cold 80% ethanol to a final concentration of 0.5 × 10^6^ cells/ml for 1 h at 4°C. The ethanol-fixed cells were centrifuged to remove ethanol and the pellet was resuspended in propidium iodide staining reagent (0.1% triton X-100, 0.1 mM EDTA, 0.05 mg/ml RNase A and 50 μg/ml propidium iodide). Cells were stored in the dark at room temperature for about 3 h. Cells were then analyzed with a flow cytometer (FC500 Beckman coulter; Beckman Coulter S.p.a., Milan, Italy) and processed by the ModFit program.

### Statistical analysis

The data are expressed as mean ± SD. Statistical analysis was carried out by paired Student’s *t*-test or ANOVA test. A p value <0.05 was considered to indicate a statistically significant difference between experimental and control groups.

## Competing interests

We declare that we have no conflict of interest.

## Authors’ contributions

CG: has made substantial contributions to conception and design, acquisition of data, analysis and interpretation of data, performed the statistical analysis, cell cultures, gene expression and cell survival assay, cell separation. PL: acquisition of data, analysis and interpretation of data, ELISA assay. DT: collection of data, has been involved in drafting the manuscript, revising it critically for important intellectual content, performed the statistical analysis, cell cultures. IB: collection of data, western blot analysis. NP: collection of data, cell cicle analysis. AC: Collection data, interpretation of data, ELISA assay. FS: has been involved in drafting the manuscript, revising it critically for important intellectual content, has given final approval of the version to be published. CC: has been involved in drafting the manuscript, revising it critically for important intellectual content, cell separation. AC: has been involved in drafting the manuscript, revising it critically for important intellectual content, performed the statistical analysis. GAP: has been involved in drafting the manuscript, revising it critically for important intellectual content, has given final approval of the version to be published. FD: has been involved in drafting the manuscript, revising it critically for important intellectual content, has given final approval of the version to be published. All authors read and approved the final manuscript.

## Pre-publication history

The pre-publication history for this paper can be accessed here:

http://www.biomedcentral.com/1471-2407/13/60/prepub
